# Differential longitudinal changes of neuronal and glial damage markers in anorexia nervosa after partial weight restoration

**DOI:** 10.1038/s41398-021-01209-w

**Published:** 2021-02-09

**Authors:** Inger Hellerhoff, Joseph A. King, Friederike I. Tam, Sophie Pauligk, Maria Seidel, Daniel Geisler, Klaas Bahnsen, Nicole Kretschmann, Katja Akgün, Veit Roessner, Tjalf Ziemssen, Stefan Ehrlich

**Affiliations:** 1 Division of Psychological and Social Medicine and Developmental Neuroscience, Faculty of Medicine, Technische Universität Dresden, Dresden, Germany; 2 Translational Developmental Neuroscience Section, Eating Disorder Research and Treatment Center, Department of Child and Adolescent Psychiatry, Faculty of Medicine, Technische Universität Dresden, Dresden, Germany; 3 Center of Clinical Neuroscience, Neurological Clinic, University Hospital Carl Gustav Carus, Faculty of Medicine, Technische Universität Dresden, Dresden, Germany; 4 Department of Child and Adolescent Psychiatry, Faculty of Medicine, University Hospital Carl Gustav Carus, Technische Universität Dresden, Dresden, Germany

**Keywords:** Biomarkers, Neuroscience, Psychiatric disorders

## Abstract

Atrophic brain changes in acute anorexia nervosa (AN) are often visible to the naked eye on computed tomography or magnetic resonance imaging scans, but it remains unclear what is driving these effects. In neurological diseases, neurofilament light (NF-L) and tau protein have been linked to axonal damage. Glial fibrillary acidic protein (GFAP) has been associated with astroglial injury. In an attempt to shed new light on factors potentially underlying past findings of structural brain alterations in AN, the current study investigated serum NF-L, tau protein, and GFAP levels longitudinally in AN patients undergoing weight restoration. Blood samples were obtained from 54 acutely underweight, predominantly adolescent female AN patients and 54 age-matched healthy control participants. AN patients were studied in the severely underweight state and again after short-term partial weight restoration. Group comparisons revealed higher levels of NF-L, tau protein, and GFAP in acutely underweight patients with AN compared to healthy control participants. Longitudinally, a decrease in NF-L and GFAP but not in tau protein levels was observed in AN patients upon short-term partial weight restoration. These results may be indicative of ongoing neuronal and astroglial injury during the underweight phase of AN. Normalization of NF-L and GFAP but not tau protein levels may indicate an only partial restoration of neuronal and astroglial integrity upon weight gain after initial AN-associated cell damage processes.

## Introduction

Anorexia nervosa (AN) is a severe eating disorder usually beginning in adolescence characterized by a persistent restriction in energy intake^1^ leading to serious medical complications^2,3^.

It has been known for decades that the brains of patients in the acutely underweight state of AN show signs compatible with atrophic changes^4^. Magnetic resonance imaging (MRI) studies have found alterations in brain structure such as substantial reductions in white and gray matter volume^5^, global cortical thinning^6^, and reduced gyrification^7^ in patients with acute AN. These alterations seem to normalize following weight gain^5,6,8^, but the mechanisms underlying these dynamic alterations are still unknown. Speculative interpretations have included apoptosis, trophic changes (smaller neurons, shorter dendrites, and fewer synapses), changes in the lipid structure of the neuronal cell wall and myelin, and the loss of astrocytes^5,6^ in acute AN.

The investigation of brain-derived proteins may aid our understanding of the underlying biology of dynamic brain changes in AN. Such proteins can be measured in cerebrospinal fluid (CSF) or in blood samples and are often associated with specific processes on cellular level^9,10^. Measurements in CSF are considered more reliable but are also more invasive. In contrast, measurements in blood have in the past been limited by the relatively lower protein concentrations, hindering reliable measurement. A promising tool to overcome these difficulties is offered by single-molecule array (Simoa) technology^11^. This method relies upon single-molecule sensitivity, reaching significantly lower limits of detection than conventional assays^11–15^ and improving reliability.

Studies in patients with neurological disorders have identified associations between specific neuronal and glial cytoskeletal proteins and different processes in the brain. Two proteins that have repeatedly been linked to axonal damage are neurofilament light (NF-L) and tau protein^16,17^. One of the most important functions of NF-L is radial cell growth^18^ while tau protein is known as a microtubule-binding protein^9^. NF-L is expressed in great amounts in large-caliber myelinated axons^10,17^, while tau protein is abundantly expressed in thin non-myelinated axons of cortical interneurons^10,19^. Elevated levels of both of these proteins have been found after brain injury^16,17^ and NF-L has also been associated with brain lesions in multiple sclerosis^17,20^. In contrast, glial fibrillary acidic protein (GFAP), an intermediate filament found in mature astrocytes^21^, which helps maintain cytoskeletal strength of glial cells is thought to be related to astroglial injury^22^ (e.g., in stroke^23^, traumatic brain injury^16^, and multiple sclerosis^24^). It is therefore commonly assumed that blood concentrations of NF-L and tau protein increase upon axonal injury^9,17,25^, whereas astrocyte injury might lead to the release of GFAP^26^. In the context of structural brain alterations in AN, such correlates of specific cell damage processes might be of particular interest.

In this study, we sought to gain a new perspective on factors potentially underlying past findings of dynamic alterations of brain structure^27^ in AN by comparing the levels of NF-L, tau protein, and GFAP of AN patients before and after short-term weight restoration (increase in body mass index (BMI) between 14% and 46%) with those of healthy control (HC) participants. Given that previous studies indicate that gray and white matter could both be affected, we expected to find elevated serum concentrations of all three brain-derived proteins in acutely underweight, predominantly adolescent female AN patients at the beginning of treatment. We were further interested in the potential consequences of the normalization of the metabolic and nutritional status in AN following partial weight restoration. We therefore compared protein levels before and after partial weight restoration, expecting to find a general reduction at the second timepoint.

## Materials and methods

### Participants

Study data were obtained from 54 underweight female patients with acute AN diagnosed according to the Diagnostic and Statistical Manual of Mental Disorders (DSM-5)^1^ and 54 pairwise age-matched female HC participants (age range 12–24 years, details in the Supplementary Information (SI) and Supplementary Figs. SF1 and SF2). One AN participant had to be excluded from the analyses because of a neurological disorder that became known after we had included that patient in our study. Consequentially, the corresponding age-matched HC participant was excluded from the study sample as well, resulting in a final sample of 53 AN and 53 HC participants. AN patients were studied in the acutely underweight state (AN_T1) not more than 96 h after admission to intensive treatment and reassessed after short-term partial weight restoration (AN_T2; after an a-priori-defined minimum BMI increase of 10%). Participants with AN underwent treatment in eating disorder programs at a tertiary care university hospital. The study was approved by the ethics committee of the Technische Universität Dresden and all participants (and the legal guardians of underage participants) gave written informed consent.

AN was diagnosed using a modified version of the German expert form of the Structured Interview for Anorexia and Bulimia Nervosa (SIAB-EX)^28^ and required a BMI < 17.5 kg/m^2^ (or below the 10th age percentile, if younger than 15.5 years). HC were recruited through advertisement among middle school, high school, and university students and had to be of normal weight, eumenorrhoeic, and without any history of psychiatric illness. Several additional exclusion criteria were applied to both AN and HC participants, including psychotropic medication in the four weeks preceding study participation (except for selective serotonin reuptake inhibitors, which were allowed in AN participants, *n* = 1 in AN_T1, *n* = 2 in AN_T2), any history of bulimia nervosa or binge eating disorder, and any medical or neurological condition that might affect appetite, eating behavior, or body weight (see SI 1.1).

Information relevant to inclusion/exclusion criteria, including lifetime neurological disorders and possible confounding variables was obtained using the SIAB-EX^28^, our own semi-structured interview, and medical records. Information about comorbid diagnoses as well as weight trajectories in the six weeks preceding study participation in AN participants was obtained from medical records and confirmed by an expert clinician.

### Clinical measures

In addition to the SIAB-EX^28^, we assessed eating disorder psychopathology using the German version of the Eating Disorder Inventory-2 (EDI-2)^29^. In line with previous reports^30–32^, we calculated a summary score representing “core” eating disorder symptoms by averaging the subscales “drive for thinness”, “body dissatisfaction”, and “bulimia”. The reason for focusing on these scales is that they represent core symptoms, a fact that can be disputed for subscales measuring e.g., feelings of ineffectiveness or maturity fears. The German version of the Beck Depression Inventory-II (BDI-II)^33^ was used to assess depressive symptoms. IQ was estimated using age-appropriate standardized instruments, mainly the Wechsler Intelligence Scales (see SI 1.2 for details). Age- and gender-corrected BMI standard deviation scores (BMI-SDS)^34,35^ were calculated from the BMI. Study data were managed using the secure, web-based electronic data capture tool REDCap^36^.

### Blood sampling and Simoa analysis

Blood sampling took place between 7 a.m. and 9 a.m. after an overnight fast and the obtained material was centrifuged after 30 min of coagulation time. The serum samples were then stored at −80 °C until further analysis. Levels of NF-L, tau protein, and GFAP were determined using the digital Simoa^TM^ Human Neurology 4-Plex A assay (item number 102153) in combination with the Simoa^TM^ HD-1 Analyzer (both Quanterix, Lexington, MA, USA) following the manufacturer’s instructions^37^. The Simoa^TM^ technology reaches significantly lower limits of detection compared to conventional assays^11,cf. 12–14^. Lower limits of detection for NF-L, tau protein, and GFAP using the Simoa^TM^ assay were 0.104, 0.024, and 0.221 pg/ml, respectively^15^. All samples were measured in duplicates, and the mean of the two measurements was used for all statistical analyses (except in one case, where only a single measurement could be obtained). The mean coefficient of variation was <10% for all three measures (NF-L: 6.92%, tau protein: 8.64%, GFAP: 3.65%).

### Statistical analyses

Statistical analyses were conducted using the software R (version 3.4.4)^38^. Extreme outliers (>3 standard deviations (SD) from the mean of the respective diagnostic group and timepoint) were excluded from all analyses resulting in the exclusion of three NF-L concentration values (*n* = 1 AN_T1, *n* = 1 AN_T2, and *n* = 1 HC), four tau protein concentration values (*n* = 1 AN_T1, *n* = 1 AN_T2, and *n* = 2 HC), and six GFAP concentration values (*n* = 3 AN_T1, *n* = 2 AN_T2, and *n* = 1 HC).

Group comparisons of brain-derived protein marker levels were conducted using false discovery rate (FDR) corrected Welch two-sample t-tests (for one-sided AN_T1 vs. HC comparisons, with correction of degrees of freedom to account for differences between groups in the variance) and matched sample t-tests (for one-sided AN_T1 vs. AN_T2 comparisons). The results of the group comparisons were validated by several additional analyses (e.g., excluding AN participants on antidepressant medication; see SI 1.3 for details). In order to validate our results against a potential violation of the normality assumption, we also reproduced the group comparisons with non-parametric tests (see SI 1.3). Since associations between NF-L and GFAP levels and age at sampling have been previously reported^12,39^, all cross-sectional results were additionally validated by linear regression analyses controlling for age and using bootstrapped 95% confidence intervals (see SI 1.3 for details).

Pearson correlations of protein levels (and change in protein levels) with BMI-SDS (as well as a change in BMI-SDS), age, BDI-II, and EDI-2 core scores were calculated within diagnostic group. Furthermore, associations between protein levels in AN_T1 and duration of illness or the weight loss in the six weeks preceding study participation were explored. All correlational analyses were validated against potential violations of normality assumptions by non-parametric correlation analyses (Kendall rank correlation coefficient, see SI 1.3 and 2.6).

## Results

### Study sample and clinical measures

The demographic and clinical characteristics of the study sample are summarized in Table 1. AN and HC participants did not differ with regard to age or IQ. AN participants had significantly lower BMI-SDS and minimal lifetime BMI and higher symptom levels than HC (EDI-2 core and BDI-II). Between timepoint one and timepoint two, AN participants showed a significant increase in BMI-SDS (range of BMI increase: 14–46%), a significant decrease in BDI-II scores, and a trend towards a decrease in EDI-2 core scores.

**Table 1:** Mean values (standard deviation) of sample characteristics and statistics of group comparisons.

	AN_T1^a^		AN_T2^b^	HC	AN_T1 vs. HC			AN_T1 vs. AN_T2		
					*t*	*df*	*p*	*t*	*df*	*p*
Age (years)	16.36 (2.29)		16.59 (2.28)	16.36 (2.24)	0.007	102.81	0.995	–	–	–
IQ		112.71 (14.13)^c^		112.13 (9.19)	0.24	79.42	0.811	–	–	–
BMI-SDS	−2.88 (0.86)		−0.67 (0.57)	−0.05 (0.70)	−18.53	98.04	<0.001***	−24.96	51	<0.001***
Minimal lifetime BMI	14.86 (1.06)		–	19.52 (1.80)	−15.51	72.94	<0.001***	–	–	–
EDI-2 core	24.57 (7.41)		23.24 (7.90)	15.82 (4.77)	7.18	86.79	<0.001***	1.77	50	0.041
BDI-II	23.47 (11.06)		15.16 (11.30)	5.98 (6.33)	9.92	80.88	<0.001***	6.30	50	<0.001***

*Note*: *AN_T1* participants with acute anorexia nervosa (mean duration of current AN episode: 10.65 months (standard deviation: 12.58)), *AN_T2* participants with anorexia nervosa after partial weight restoration, *HC* healthy control participants, *BMI-SDS* body mass index standard deviation score, *EDI-2* Eating Disorder Inventory, version 2; *BDI-II* Beck Depression Inventory, version 2.

^a^44 AN participants were of the restrictive and nine of the binge-eating/purging subtype.

^b^Mean duration between assessments in AN patients was 81.57 days (range: 35–154).

^c^IQ was only measured once in AN participants after weight gain.

***False discovery rate (FDR) < 0.001.

### Analysis of brain-derived protein markers

Group comparisons revealed significantly increased levels of NF-L, tau protein, and GFAP in AN_T1 compared to HC. All statistics are reported in Table 2. Linear regression analyses controlling for age confirmed these results (see SI 2.1 for details). In the regression analyses, GFAP was significantly predicted by diagnostic group, age, and the interaction of group with age (see SI, Table S1 for details). A visual inspection of these results (SI Fig. SF3) also revealed that the age effect in GFAP levels was mainly driven by acAN participants (see also correlational analysis below).

**Table 2:** Mean values (standard deviation) of brain-derived protein marker concentrations and statistics of group comparisons.

	AN_T1	AN_T2	HC	AN_T1 vs. HC			AN_T1 vs. AN_T2		
				*t*	*df*	*p*	*t*	*df*	*p*
NF-L (pg/ml)	11.99 (6.99)	7.08 (4.52)	6.15 (2.53)	5.66	64.15	<0.001***	7.45	51	<0.001***
Tau (pg/ml)	1.50 (0.72)	1.50 (0.68)	1.18 (0.58)	2.53	97.35	0.006**	0.03	51	0.487
GFAP (pg/ml)	136.33 (44.66)	106.43 (32.65)	98.48 (27.79)	5.12	81.44	<0.001***	7.73	49	<0.001***

*Note*: *AN_T1* participants with acute anorexia nervosa, *AN_T2* participants with anorexia nervosa after partial weight restoration, *HC* healthy control participants, *NF-L* neurofilament light, *GFAP* glial fibrillary acidic protein.

**False discovery rate (FDR) < 0.01.

***FDR < 0.001.

In line with the results of the regression analyses, two-sided correlation analyses revealed a negative association between GFAP and age in AN_T1 (*r* = −0.415; *p* = 0.003). No other significant correlations between brain-derived protein concentrations and age, BMI-SDS, EDI-2 core, or BDI-II scores were found (SI Table S2). However, GFAP (and on a trend level NF-L) but not tau protein levels in AN_T1 were positively associated with the amount of weight loss in the six weeks prior to blood sampling (one-sided analyses; GFAP: *r* = 0.375; *p* = 0.008; NF-L: *r* = 0.234; *p* = 0.068). None of the proteins were related to duration of illness in AN_T1 (SI Table S3).

In AN participants, there was a significant decrease from the first to the second measurement in NF-L and in GFAP levels, but not in tau protein levels (all statistics reported in Table 2). No differences were found between AN_T2 and HC participants in NF-L and GFAP levels (see SI 2.5).

Change in NF-L and GFAP levels was significantly associated with change in BMI-SDS (one-sided analyses; NF-L: *r* = −0.417; *p* = 0.001; GFAP: *r* = −0.361; *p* = 0.005), while the change in tau protein levels was not (see SI Table S4).

The results from both cross-sectional and longitudinal group comparisons were confirmed by analyses excluding AN participants on antidepressant medication (*n* = 1 in AN_T1, *n* = 2 in AN_T2), or excluding AN participants of the binge-eating/purging subtype (*n* = 9; see SI 2.2 and 2.3 and Supplementary Table S5 for details). Furthermore, all results from the group comparisons and correlational analyses were confirmed by non-parametric analyses as described above (SI Tables S6–S9). The only exception was the association between GFAP and age in AN_T1, which did not survive FDR-correction in the nonparametric approach.

## Discussion

The aim of this study was to gain insight into factors potentially underlying the well-replicated and substantial brain structure alterations in AN^5–7,40^ by longitudinally studying brain-derived neural and glial protein markers. We found elevated levels of NF-L, tau protein, and GFAP in acutely underweight AN patients at the beginning of weight restoration treatment compared to HC. These findings may be indicative of neuronal and glial damage processes in the acute phase of AN^10,17,22^. Upon partial weight restoration, NF-L and GFAP levels normalized, but tau protein levels remained elevated. Since tau protein is abundantly found in thin non-myelinated axons of cortical interneurons^10,19^, this finding might indicate that neuronal damage processes in these cell types persist upon short-term/partial weight restoration.

Neurofilaments are structural scaffolding proteins exclusively expressed in neurons^41^. The most abundant subunit of neurofilaments is NF-L^17^. It mainly occurs in axons, where it is responsible for radial growth and thus aids to increase conduction velocity^18^. Upon axonal injury, NF-L concentration levels increase significantly in blood and may therefore be considered a damage marker^17^. In our study, we found elevated serum levels of NF-L in AN_T1 compared to HC. This finding suggests that axons may be damaged in the acutely underweight phase of AN. These results are in line with the only previous study examining NF-L levels in acute AN using a cross-sectional design and single-marker assay^39^. Following short-term partial weight restoration, we observed a normalization of NF-L levels in AN participants, which was associated with the increase in BMI-SDS. This may reflect a cessation of axonal damage processes. Similar observations have been made in patients with multiple sclerosis, where a reduction of NF-L levels was seen after clinically effective treatment^42^.

Tau protein levels were also elevated in AN_T1 compared to HC. Tau is a microtubule-binding protein^9^. In the brain, it is mainly found in neurons, where it is primarily localized in axons^43,44^. Its functions include the stabilization of microtubules, promoting microtubule assembly, and the regulation of axonal transport^44^. Tau protein—like NF-L—is hypothesized to be an axonal damage marker because concentrations are increased in CSF and blood following injury^9,10,25^. Elevated tau protein levels have been detected in blood samples of populations suffering from various forms of brain injury (e.g., traumatic brain injury, concussion, and hypoxic brain injury^16,25,45^). Our finding of elevated tau protein levels in AN_T1 thus substantiates the hypothesis of axonal injury occurring in the acutely underweight phase of AN.

Tau protein levels—unlike NF-L and GFAP levels—did not decrease upon short-term partial weight restoration. To our knowledge, this is the first study investigating tau protein levels in AN. A replication of this finding by other studies is therefore needed. If confirmed, a possible explanation might be that the duration of cell damage processes varies in different types of neurons. Tau protein can be found in high concentrations in thin non-myelinated axons of cortical interneurons^10,19^, while NF-L is abundant in large-caliber myelinated axons^10,17^. It is thus tempting to speculate that whereas myelinated axons might be rapidly restored after weight gain, cortical interneurons could remain affected by ongoing axonal injury over longer time periods. GFAP (and on a trend level NF-L) but not tau protein levels in AN_T1 were associated with weight loss in the six weeks before study participation. This, too, hints that the release dynamics of tau protein might be different than for NF-L and GFAP. To answer this question, it might be insightful to study whether tau protein levels normalize slower than NF-L and GFAP levels or if they remain elevated even in long-term fully (weight-)recovered individuals with a history of AN (recAN). To our knowledge, tau protein levels in recAN have not yet been investigated, but Nilsson et al.^39^ reported higher NF-L levels in recAN than in HC. The discrepancy to our results could potentially be due to the fact that the present study included predominantly adolescent AN participants, while the recAN studied by Nilsson et al.^39^ were older. A recent neuroimaging study^27^ showed that the restoration of cortical thickness in AN patients is negatively correlated with age, suggesting slower brain restitution in older patients – potentially as a result of decreased brain plasticity.

GFAP is an intermediate filament found in mature astrocytes^21^. It is responsible for the cytoskeletal structure of glial cells and helps to maintain their mechanical strength^26^. Elevated GFAP levels have been associated with astroglial injury and cell death and normalization of GFAP levels is hypothesized to reflect the restoration of astroglial integrity^22^. Hence, our findings of elevated GFAP levels in AN_T1 suggest that glial damage may occur in the acutely underweight AN state. Astroglial integrity seems to be restored after partial weight restoration as indicated by the normalization of GFAP levels observed in AN_T2. Also the reduction of GFAP levels—like NF-L—was associated with the increase in BMI-SDS. To date only one other study has studied GFAP levels in patients with acute AN: Ehrlich et al.^12^ did not find any significant difference in GFAP levels between acutely underweight AN patients and HC. A possible explanation for these different findings might be the use of the highly sensitive Simoa technology in the present study, allowing for the detection of smaller differences in protein concentrations. We found a (trend towards a) negative association between GFAP and age in AN_T1, which was not seen in HC. This finding is in line with the significant interaction seen in the regression analysis predicting GFAP. Given that GFAP levels usually increase with age, a positive association between age and GFAP levels would have been expected^21,46^. GFAP has been identified as an astrocyte maturation marker because astrocyte maturation coincides with a switch between vimentin and GFAP expression^47^. The finding of a negative correlation in AN_T1 might thus point towards disrupted maturational processes in acutely underweight AN patients^12^—potentially due to undernutrition.

Overall, the findings of elevated NF-L, tau protein, and GFAP levels in AN_T1 are thus suggestive of ongoing damage to brain cells. Although we did not analyze potential relationships with measures of brain structure, these findings are generally in line with previous structural MRI findings of white and gray matter atrophy and cortical thinning in patients with acute AN^5,6^. Elevated NF-L and tau protein levels point towards damage of neuronal axons. The rapid normalization of NF-L and GFAP levels after weight gain may reflect a return to (partially) normal cellular functioning in the brain. This, again, is in line with neuroimaging studies showing that brain structure alterations normalize upon partial weight restoration^5,8^.

Regarding the mechanisms underlying brain structure alterations in acute AN, several hypotheses have been put forward^5,6^. An early hypothesis has been the degeneration of neurons^6,48,49^. Actual cell death could explain the elevated NF-L and tau protein levels in acutely underweight AN. However, given the relatively fast normalization of gray and white matter reductions with weight restoration in AN, classical neurodegeneration with following neurogenesis is unlikely to be the reason for brain structure alterations in AN^6^. Human adult neurogenesis, where present, proceeds at turnover rates not compatible with the speed of brain volume normalizations observed in AN (e.g., <2% median annual turnover in hippocampal neurons)^50^. It is, therefore, more reasonable to assume that a damage process without complete degeneration of neurons takes place. Based on post-mortem case studies and theoretical considerations these remodeling processes have been hypothesized to include changes to dendrites (shorter dendrites, less dendritic branching, fewer spines, and dendritic spine abnormalities) as well as soma (changes to soma size and density)^5,6,51^. Rodent studies have yielded mixed results regarding neuronal alterations and/or damage in preclinical models of acute AN. In the activity-based anorexia (ABA) animal model, Frintrop et al.^52^ found no alteration in neuron number and size. On the other hand, Chowdhury et al.^53^ found morphological changes in CA1 pyramidal cells in the hippocampus in ABA. Other rodent studies have found pre- and/or postnatal protein malnutrition to be associated with a reduced number of neurons and a decrease in the diameter of apical dendrites of hippocampal pyramidal cells^54,55^.

The elevated GFAP levels found in AN_T1 suggest astrocyte injury. This finding, too, has to be interpreted in the light of the brain volume normalizations that were reported in association with weight restoration in AN^5^. Complete degeneration of astrocytes would thus be plausible only if astrocytes could be rapidly regenerated upon weight gain. Astrocyte turnover in the healthy brain is slow and there is only little proliferation or new generation of astrocytes^56^. However, upon injury of the central nervous system (CNS), reactive astrogliosis can occur, associated with different processes depending on the severity of the CNS injury^56^. Reactive astrogliosis can lead to an upregulation of GFAP expression and the proliferation of astrocytes which could make this a potential mechanism for the regeneration of astrocytes. However, this process seems unlikely to underlie protein level and brain structure normalizations observed in AN for various reasons. For example, severe diffuse reactive astrogliosis is usually observed in reaction to severe local CNS impairments different from the general brain atrophy seen in acute AN. Moreover, if astrogliosis occurred in AN, one would expect it to begin early during weight loss and ongoing brain damage and not upon weight gain. All in all, it therefore seems likely that GFAP is not released due to astrocyte degeneration but rather due to astrocyte damage or alterations (e.g., smaller or less mature astrocytes^6^). Astrocytes interact with neurons in multiple ways: For example, they play an important role in synaptic transmission and in CNS metabolism^56^. Astrocyte damage could therefore be directly linked to neuronal function and maybe even to neuronal injury. The importance of astrocyte alterations in AN is also underscored by findings in animal models of AN^52,57^. In the ABA paradigm^52^, for example, brain volume reductions after starvation were shown to be associated with a reduction in GFAP-stained astrocytes, a reduced mean astrocyte cell surface, and a reduction in the mean number of proliferating cells. After refeeding, these changes were no longer evident^52^.

Associations of NF-L, tau, and/or GFAP levels with the severity of brain damage or brain atrophy have also been found in conditions such as traumatic brain injury, stroke, Alzheimer’s disease, and old age^9,16,23,58,59^. These findings strengthen our hypothesis of subtle neuronal and glial damage underlying the elevated brain-derived protein marker levels found in acutely underweight AN patients. Others have reported associations of NF-L, tau protein, and GFAP levels with cognitive impairments in various diseases or in healthy individuals^58,60–64^. Future studies should test such relationships in the context of AN, especially since impairments in cognitive functioning in AN have been reported^65,66^.

The results of our study have to be considered in light of several limitations. First, we measured protein concentrations in blood samples. While this method has the advantage of being less invasive than CSF sampling, it has been hypothesized that the blood–brain–barrier might be more permeable in acute AN^5,39^. In the context of our results, the notion that astrocytes are likely to be relevant for maintaining the blood-brain-barrier^56,67^ makes this point particularly relevant. The increased serum protein marker levels observed in this study could thus be due to increased blood–brain–barrier permeability rather than caused by an actual increase in the release from brain cells. Studies of protein marker levels in CSF of AN patients are needed to address this problem. Furthermore, the peripheral brain-derived protein concentrations may be influenced by inflammatory and hormonal changes in AN. Second, since our study group consisted mainly of adolescent participants, a bias due to developmental effects could be present. This bias is, however, unlikely to have influenced the results of our group comparisons, since pairwise age-matching was carried out resulting in very small age differences in single pairs. Further studies are however needed to explore NF-L, tau protein, and GFAP levels in older AN patients. Since the speed of normalization of structural brain changes in AN may depend on age^27^, age-related effects on protein levels as well might be found in study samples with a broader age range. Third, another limitation of the study is the naturalistic convenience sample, the lack of premorbid data in AN, and the absence of longitudinal protein level measurements in HC. Future studies should also strive to collect objective data on weight trajectories and dietary intake preceding blood sampling in order to avoid self-report-biases. Fourth, to test the utility of NF-L, tau protein, and GFAP as prognostic or monitoring markers, a prolonged sampling (perhaps in combination with MRI data collection) in individuals cycling through the stages of relapse and remission is needed. Last but not least, it has to be noted that we did not explore associations with brain macrostructure. Conclusions regarding how the current findings might relate to previous MRI findings must thus be drawn with care and are necessarily speculative. Future studies should focus on this question by directly exploring associations of brain-derived protein markers with brain macrostructure in patients with neuropsychiatric disorders as well as healthy individuals. The present work has multiple strengths, including being the first study to simultaneously assess three well-established brain-derived protein markers of brain injury in AN using the highly sensitive Simoa technology. Furthermore, the collection of longitudinal data allows for a distinction between effects in the acute undernourished state and effects outlasting partial weight restoration, as seen in the differential temporal profiles of NF-L and tau protein levels.

To conclude, we found elevated NF-L, GFAP, and tau protein serum levels in acutely underweight AN participants suggesting ongoing neuronal and glial damage in the brain. Relatively fast normalization of NF-L and GFAP levels after weight gain may indicate a partial restoration of neuronal and glial integrity. The persistence of elevated tau protein may suggest that the damage processes to thin non-myelinated axons of cortical interneurons may persist over a longer period of time. Further research in this field might promote the understanding of processes occurring on a neuronal and glial level in AN and thus help to identify key targets for the development of new therapeutic interventions that may also include neuroprotective pharmacological agents^68,69^.

## Supplementary information

Supplementary Information
