# Supplementary material for: Differential longitudinal changes of neuronal and glial damage markers in anorexia nervosa after partial weight restoration
Source: Transl Psychiatry. 2021 Feb 9;11:86. doi: 10.1038/s41398-021-01209-w (PMC7870648; doi:10.1038/s41398-021-01209-w)
Supplement: Supplementary file 1 — Supplementary Information [file 41398_2021_1209_MOESM1_ESM.docx]

# Supplementary Information

## 1. Supplementary Methods

### 1.1 Participants

Healthy control participants (HC) were pairwise age-matched to patients with acute anorexia nervosa (AN) using an implementation of the Munkres algorithm^1^. The mean age difference between one pair of participants was 0.06 years (maximum: 0.7 years). Histograms of age in AN_T1 and HC participants are presented in Supplementary Figures SF1 and SF2. Due to missing effect sizes regarding neurofilament light (NF-L), tau protein, and glial fibrillary acidic protein (GFAP) levels in AN, studies on other neurological or psychiatric disorders were consulted to assure the adequacy of the sample size^2–5^. This could be clearly determined for NF-L and GFAP, while further studies are needed regarding tau protein. Normal weight in HC was defined by a body mass index (BMI) between 18.5 kg/m^2^ and 28 kg/m^2^ (or between the 10^th^ and the 94^th^ age percentile, in participants younger than 19 years). Additional exclusion criteria for HC were a lifetime BMI below 17.5 kg/m^2^ (or below the 10^th^ age percentile when younger than 19 years) and substantial weight loss in the four weeks preceding study participation. AN were included if no substantial weight gain was reported in the four weeks preceding the first study point. In addition to the exclusion criteria mentioned in the main manuscript, participants in both groups were excluded if they had any history of organic brain syndrome, dementia, schizophrenia, psychosis, bipolar disorder, or substance dependence. Further exclusion criteria for all participants were an IQ below 85, current substance abuse, current inflammatory, neurologic, or metabolic illness, clinically relevant anemia, pregnancy, and breast feeding.

### 1.2 Clinical measures

IQ was estimated using full or short versions of age-appropriate standardized instruments, mainly German versions of the Wechsler Intelligence Scales for adults or children (HAWIK-IV^6^ in *n* = 60; WISC-IV^7^ in *n* = 1; WISC-V^8^ in *n* = 2; WIE^9^ in *n* = 33; WAIS-IV^10^ in *n* = 5). Short versions were used in 59 participants. In four cases, IQ was missing.

### 1.3 Statistical analyses

Since previous studies have reported associations between NF-L as well as GFAP levels and age at sampling^11,12^, all cross-sectional results were validated by linear regression analyses controlling for age. For each protein marker, two regression models were constructed: In the first model (model A), we predicted protein marker concentration levels using diagnostic group and age at sampling as predictors. In the second model (model B), diagnostic group, age at sampling, and an interaction term of diagnostic group and age at sampling were included as predictors. For each protein marker, the best model was selected using the Akaike information criterion (AIC)^13^. To validate these regression analyses against potential slight violations of normal distribution, we calculated 95% confidence intervals (CI) using bootstrapping (2000 replications).

To control for potential effects of AN subtype or antidepressant medication in AN, all main cross-sectional and longitudinal analyses were repeated twice: excluding AN participants on antidepressant medication (*n* = 1 in AN_T1, *n* = 2 in AN_T2) and excluding AN participants of the binge-eating/purging subtype (*n* = 9) from the analyses. In order to validate our results against potential violation of normality assumptions, we also repeated the group comparisons and all correlational analyses with non-parametric tests. For cross-sectional group comparisons, one-sided Wilcoxon rank sum tests were carried out. Longitudinal group comparisons were validated by one-sided Wilcoxon signed rank tests. Correlational analyses were repeated using Kendall rank correlation coefficients.

Additional exploratory two sample Welch t-tests (two-sided) were conducted to compare concentration levels of NF-L, tau protein, and GFAP between AN_T2 and HC.

## 2. Supplementary Results

### 2.1 Regression analyses controlling for age

We confirmed the results of the cross-sectional group comparisons by regression analyses controlling for age. For NF-L and tau protein, the best model according to the AIC was model A, including group and age at sampling but not their interaction as predictors. For GFAP, the best model was model B, additionally including the interaction term between diagnostic group and age at sampling. Results of the regression analyses are summarized in Table S1. All results were additionally supported by the bootstrapped CIs (Table S1). The regression analysis for GFAP yielded significant effects of study group, age, and the interaction of study group with age. This interaction is plotted in the Supplementary Figure SF3 and discussed in the main manuscript.

### 2.2 Replication of group comparisons with exclusion of participants on antidepressant medication

Exclusion of AN participants on antidepressant medication (*n* = 1 in AN_T1, *n* = 2 in AN_T2) did not change the results of the group comparisons. Statistics are reported in Table S5.

### 2.3 Replication with exclusion of AN participants of the binge-eating/purging subtype

Exclusion of AN participants of the binge-eating/purging subtype (*n* = 9) did not change the results of the group comparisons. Statistics are reported in Table S5.

### 2.4 Non-parametric replication of group comparisons

Non-parametric group comparisons confirmed the results reported in the main manuscript. They revealed significantly increased levels of NF-L, tau protein, and GFAP in AN_T1 compared to HC. In AN participants, the significant decrease from the first to the second measurement in NF-L and in GFAP levels, but not in tau protein levels were confirmed (all statistics reported in Table S6).

### 2.5 Comparison of brain-derived protein marker levels between AN_T2 and HC participants

Tau protein levels were significantly higher in AN_T2 than in HC participants (*t*(99.35) = 2.61; *p* = .010). No difference was detected between AN_T2 and HC participants in NF-L (*t*(80.15) = 1.29; *p* = .201) or GFAP levels (*t*(97.86) = 1.33; *p* = .187).

### 2.6 Correlation analyses

Two-sided Pearson correlations of protein marker levels with age, body mass index standard deviation scores (BMI-SDS), EDI-2 core scores (average of the the subscales “drive for thinness”, “body dissatisfaction”, and “bulimia” of the Eating Disorder Inventory, version 2), and Beck Depression Inventory, version 2 (BDI-II) scores were calculated. All results are reported in Table S2. In the non-parametric analyses (Table S7) the correlation between GFAP levels in AN_T1 and age did not survive FDR-correction.

Additional one-sided Pearson correlation analyses were performed to test for associations between protein levels in AN_T1 and duration of illness or the weight trajectory in the six weeks preceding study participation. We also tested for associations between change in protein levels and change in BMI-SDS in AN participants (one-sided Pearson correlation). The results of these parametric analyses are reported in the supplementary Tables S3 and S4. The results of the non-parametric correlation analyses are shown in the supplementary Tables S8 and S9.

# References for the Supplementary Information

1. Munkres, J. Algorithms for the assignment and transportation problems. *J. Soc. Ind. Appl. Math.* **5**, 32–38 (1957).

2. Rohrer, J. D. *et al.* Serum neurofilament light chain protein is a measure of disease intensity in frontotemporal dementia. *Neurology* **87**, 1329–1336 (2016).

3. Weston, P. S. J. *et al.* Serum neurofilament light in familial Alzheimer disease: A marker of early neurodegeneration. *Neurology* **89**, 2167–2175 (2017).

4. Evered, L., Silbert, B., Scott, D. A., Zetterberg, H. & Blennow, K. Association of changes in plasma neurofilament light and tau levels with anesthesia and surgery. *JAMA Neurol.* **75**, 542–547 (2018).

5. Wang, J., Zou, Q., Han, R., Li, Y. & Wang, Y. Serum levels of Glial fibrillary acidic protein in Chinese children with autism spectrum disorders. *Int. J. Dev. Neurosci. Off. J. Int. Soc. Dev. Neurosci.* **57**, 41–45 (2017).

6. Petermann, F. & Petermann, U. *HAWIK-IV. Hamburg-Wechsler-Intelligenztest für Kinder IV*. (Huber, 2007).

7. Petermann, F. & Petermann, U. *WISC-IV. Wechsler Intelligence Scale for Children - Fourth Edition*. (Pearson Assessment & Information, 2011).

8. Petermann, F. *WISC-V. Wechsler Intelligence Scale for Children - Fifth Edition*. (Pearson Assessment & Information, 2017).

9. von Aster, M., Neubauer, A. & Horn, R. *WIE. Wechsler Intelligenztest für Erwachsene*. (Pearson Assessment & Information, 2008).

10. Petermann, F. *WAIS-IV. Wechsler adult intelligence scale*. (Pearson Assessment & Information, 2012).

11. Ehrlich, S. *et al.* Glial and neuronal damage markers in patients with anorexia nervosa. *J. Neural Transm.* **115**, 921–927 (2008).

12. Nilsson, I. A. K. *et al.* Plasma neurofilament light chain concentration is increased in anorexia nervosa. *Transl. Psychiatry* **9**, 180 (2019).

13. Akaike, H. A new look at the statistical model identification. *IEEE Trans. Autom. Control* **19**, 716–723 (1974).

Supplementary Figure SF1:

*Age distribution in AN_T1 participants*

*
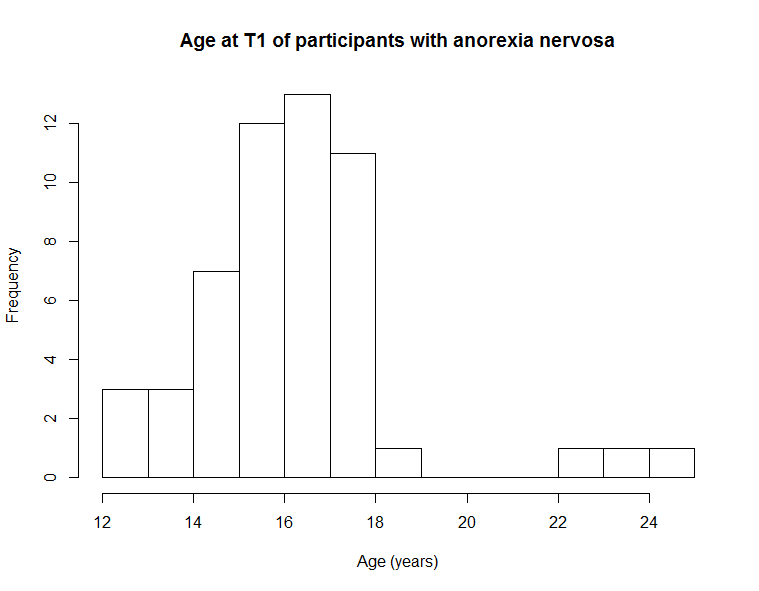
*

Supplementary Figure SF2:

*Age distribution in HC participants*

*
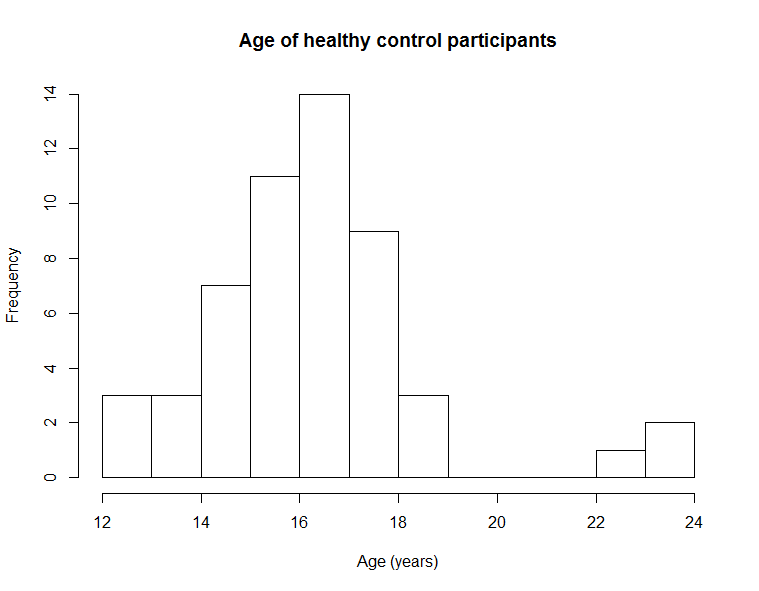
*

Supplementary Figure SF3:

*Age and GFAP levels in AN_T1 and HC participants with regression lines and 95% confidence interval*

*
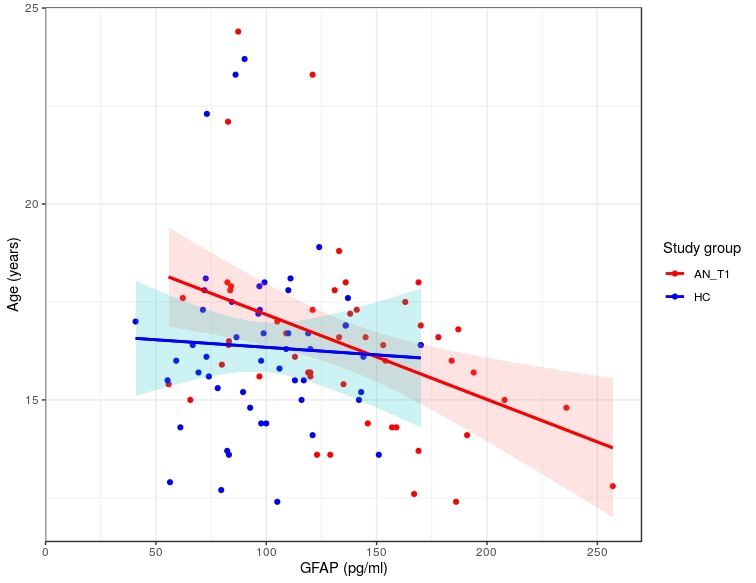
*

*Note.* GFAP = glial fibrillary acidic protein; AN_T1 = participants with anorexia nervosa; HC = healthy control participants.

Table S1:

Summary (b-values (B), standard errors of b-values (SE B), significance levels (p), and bootstrapped 95% confidence intervals (bootCI)) of regression analyses predicting brain-derived protein marker levels from diagnostic group and age at sampling

|  | ***B*** | ***SE B*** | ***p*** | ***95% bootCI^1^*** |
| --- | --- | --- | --- | --- |
| **NF-L** |  |  |  |  |
| **Group** | -5.838 | 1.035 | < 0.001*** | [-8.141, -4.001] |
| **Age** | -0.128 | 0.230 | 0.579 | [-0.421, 0.173] |
| **Tau** |  |  |  |  |
| **Group** | -0.327 | 0.130 | 0.013* | [-0.602, -0.078] |
| **Age** | -0.023 | 0.029 | 0.421 | [-0.073, 0.027] |
| **GFAP** |  |  |  |  |
| **Group** | -158.802 | 50.470 | 0.002** | [-265.8, -78.1] |
| **Age** | -7.961 | 2.147 | < 0.001*** | [-14.264, -4.336] |
| **Group*Age** | 7.377 | 3.054 | 0.018* | [2.678, 13.713] |

*Note.* NF-L = neurofilament light; GFAP = glial fibrillary acidic protein.

^1^Bootstrapping performed with 2000 replications. **p* < .05. ***p* < .01. ****p* < .001.

Table S2:

Correlation coefficients (r) and significance levels (p) of Pearson correlations between brain-derived protein marker levels and demographic/clinical measures

|  |  | **Age** | | **BMI-SDS** | | **EDI-2 core** | | **BDI-II** | |
| --- | --- | --- | --- | --- | --- | --- | --- | --- | --- |
|  |  | ***r*** | ***p*** | ***r*** | ***p*** | ***r*** | ***p*** | ***r*** | ***p*** |
| **AN_T1** | **NF-L** | -0.123 | 0.384 | -0.238 | 0.090 | 0.052 | 0.717 | 0.260 | 0.063 |
|  | **Tau** | -0.136 | 0.338 | -0.038 | 0.791 | 0.027 | 0.849 | 0.101 | 0.477 |
|  | **GFAP** | -0.415 | 0.003* | -0.020 | 0.891 | -0.028 | 0.847 | 0.037 | 0.798 |
| **HC** | **NF-L** | 0.114 | 0.423 | -0.198 | 0.159 | - | - | - | - |
|  | **Tau** | -0.010 | 0.943 | -0.083 | 0.564 | - | - | - | - |
|  | **GFAP** | -0.047 | 0.738 | -0.310 | 0.025 | - | - | - | - |

*Note.* BMI-SDS = body mass index standard deviation score; EDI-2 = Eating Disorder Inventory, version 2; BDI-II = Beck Depression Inventory, version 2; AN_T1 = participants with anorexia nervosa; HC = healthy control participants; NF-L = neurofilament light; GFAP = glial fibrillary acidic protein.

* False discovery rate (FDR) < .05.

Table S3:

One-sided tests for correlations between brain-derived protein markers in AN_T1 participants and duration of illness and weight loss in the six weeks preceding the first blood sampling

|  | **Duration of illness** | | **Weight loss in six weeks before blood sampling** | |
| --- | --- | --- | --- | --- |
|  | ***r*** | ***p*** | ***r*** | ***p*** |
| **NF-L in AN_T1** | -0.180 | 0.894 | 0.234 | 0.068 |
| **Tau protein in AN_T1** | -0.076 | 0.699 | 0.031 | 0.423 |
| **GFAP in AN_T1** | -0.307 | 0.983 | 0.375 | 0.008* |

*Note.* AN_T1 = participants with acute anorexia nervosa ; NF-L = neurofilament light; GFAP = glial fibrillary acidic protein. *False Discovery Rate (FDR) < 0.05.

Table S4:

One-sided tests for correlations between change in brain-derived protein marker levels and change in BMI-SDS in participants with anorexia nervosa

|  | **Change in BMI-SDS** | |
| --- | --- | --- |
|  | ***r*** | ***p*** |
| **Change NF-L** | -0.417 | 0.001** |
| **Change tau protein** | -0.120 | 0.198 |
| **Change GFAP** | -0.361 | 0.005** |

*Note*. BMI-SDS = body mass index standard deviation score; NF-L = neurofilament light; GFAP = glial fibrillary acidic protein. **False Discovery Rate (FDR) < 0.01.

Table S5:

Test statistics of group comparisons of brain-derived protein marker levels (A) excluding AN participants on antidepressant medication and (B) excluding AN participants of the binge-eating/purging subtype

|  |  | 1. **Replication with exclusion of AN participants on antidepressant medication** | | | 1. **Replication with exclusion of AN participants of the binge-eating/purging subtype** | | |
| --- | --- | --- | --- | --- | --- | --- | --- |
|  |  | *t* | *df* | *p* | *t* | *df* | *p* |
| **AN_T1 vs. HC** | **NF-L** | 5.53 | 62.45 | < 0.001*** | 5.49 | 50.84 | < 0.001*** |
|  | **Tau** | 2.62 | 95.62 | 0.005** | 3.20 | 73.76 | 0.001** |
|  | **GFAP** | 5.25 | 79.72 | < 0.001*** | 4.76 | 62.13 | < 0.001*** |
| **AN_T1 vs. AN_T2** | **NF-L** | 7.28 | 49 | < 0.001*** | 6.81 | 42 | < 0.001*** |
|  | **Tau** | 0.08 | 49 | 0.468 | 0.07 | 42 | 0.472 |
|  | **GFAP** | 7.61 | 47 | < 0.001*** | 8.15 | 40 | < 0.001*** |

*Note.* AN = anorexia nervosa; AN_T1 = participants with anorexia nervosa; HC = healthy control participants; AN_T2 = participants with anorexia nervosa after partial weight restoration; NF-L = neurofilament light; GFAP = glial fibrillary acidic protein.

**False discovery rate (FDR) < .01. ***FDR < .001.

Table S6:

Statistics of non-parametric group comparisons of brain-derived protein marker concentrations

|  | **AN_T1 vs. HC** | | **AN_T1 vs. AN_T2** | |
| --- | --- | --- | --- | --- |
|  | ***W*** | ***p*** | ***V*** | ***p*** |
| **NF-L (pg/ml)** | 2249 | < 0.001*** | 1354 | < 0.001*** |
| **Tau (pg/ml)** | 1735 | 0.004** | 671.5 | 0.565 |
| **GFAP (pg/ml)** | 1962 | < 0.001*** | 1212 | < 0.001*** |

*Note.* AN_T1 = participants with acute anorexia nervosa; HC = healthy control participants; AN_T2 = participants with anorexia nervosa after partial weight restoration; NF-L = neurofilament light; GFAP = glial fibrillary acidic protein.

**False discovery rate (FDR) < .01. ***FDR < .001.

Table S7:

Kendall’s tau and significance levels (p) of two-sided Kendall rank correlations between brain-derived protein marker levels and demographic/clinical measures

|  |  | **Age** | | **BMI-SDS** | | **EDI-2 core** | | **BDI-II** | |
| --- | --- | --- | --- | --- | --- | --- | --- | --- | --- |
|  |  | ***Kendall’s tau*** | ***p*** | ***Kendall’s tau*** | ***p*** | ***Kendall’s tau*** | ***p*** | ***Kendall’s tau*** | ***p*** |
| **AN_T1** | **NF-L** | -0.061 | 0.527 | -0.220 | 0.022 | 0.041 | 0.670 | 0.167 | 0.085 |
|  | **Tau** | -0.134 | 0.167 | -0.056 | 0.559 | -0.019 | 0.844 | 0.030 | 0.758 |
|  | **GFAP** | -0.256 | 0.009 | -0.043 | 0.664 | 0.022 | 0.821 | -0.016 | 0.874 |
| **HC** | **NF-L** | 0.122 | 0.204 | -0.146 | 0.126 | - | - | - | - |
|  | **Tau** | -0.018 | 0.852 | -0.103 | 0.287 | - | - | - | - |
|  | **GFAP** | 0.002 | 0.987 | -0.209 | 0.029 | - | - | - | - |

*Note.* BMI-SDS = body mass index standard deviation score; EDI-2 = Eating Disorder Inventory, version 2; BDI-II = Beck Depression Inventory, version 2; AN_T1 = participants with anorexia nervosa; HC = healthy control participants; NF-L = neurofilament light; GFAP = glial fibrillary acidic protein.

Table S8:

One-sided non-parametric tests for correlations between brain-derived protein markers in AN_T1 participants and duration of illness and weight loss in the six weeks preceding the first blood sampling

|  | **Duration of illness** | | **Weight loss in six weeks before blood sampling** | |
| --- | --- | --- | --- | --- |
|  | ***Kendall’s tau*** | ***p*** | ***Kendall’s tau*** | ***p*** |
| **NF-L in AN_T1** | -0.073 | 0.769 | 0.261 | 0.008* |
| **Tau protein in AN_T1** | -0.033 | 0.631 | 0.041 | 0.352 |
| **GFAP in AN_T1** | -0.220 | 0.985 | 0.238 | 0.015* |

*Note.* AN_T1 = participants with acute anorexia nervosa; NF-L = neurofilament light; GFAP = glial fibrillary acidic protein. *False Discovery Rate (FDR) < 0.05.

Table S9:

One-sided non-parametric tests for correlations between change in brain-derived protein marker levels and change in BMI-SDS in participants with anorexia nervosa

|  | **Change in BMI-SDS** | |
| --- | --- | --- |
|  | ***Kendall’s tau*** | ***p*** |
| **Change NF-L** | -0.264 | 0.003** |
| **Change tau protein** | -0.071 | 0.229 |
| **Change GFAP** | -0.252 | 0.005** |

*Note.* BMI-SDS = body mass index standard deviation score; NF-L = neurofilament light; GFAP = glial fibrillary acidic protein. **False Discovery Rate (FDR) < 0.01
